# Supplementary material for: Characterization of an Mtbp Hypomorphic Allele in a Diethylnitrosamine-Induced Liver Carcinogenesis Model
Source: Cancers (Basel). 2023 Sep 16;15(18):4596. doi: 10.3390/cancers15184596 (PMC10526184; doi:10.3390/cancers15184596)

Figure 1

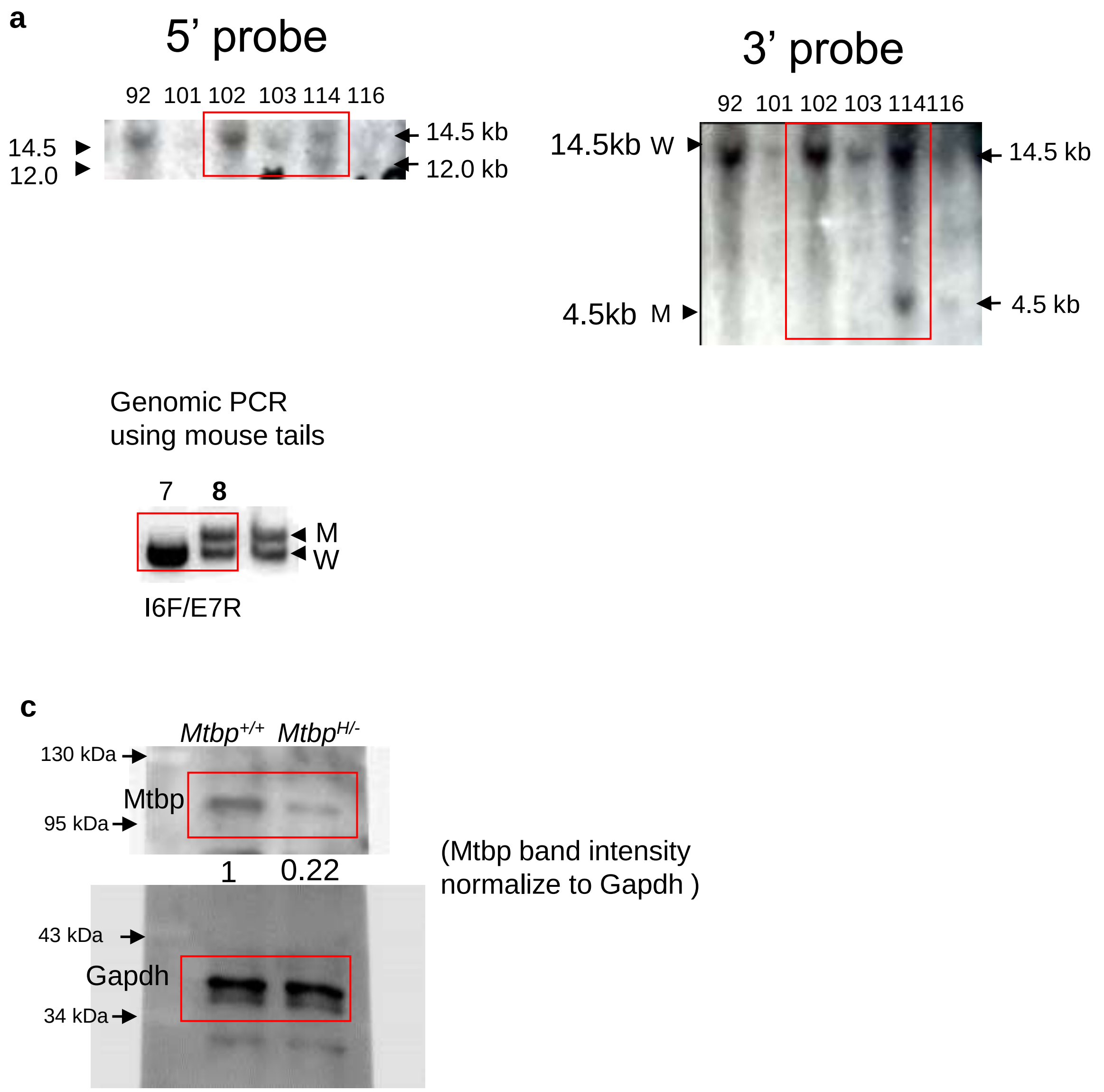

a
5’ probe
92 101 102 103 114 116
14.5
12.0
14.5 kb
12.0 kb
3’ probe
92 101 102 103 114116
14.5kb W
4.5kb M
14.5 kb
4.5 kb
Genomic PCR
using mouse tails
7 8
M
W
I6F/E7R
c
Mtbp+/+ MtbpH/-
130 kDa
Mtbp
95 kDa
1 0.22
43 kDa
Gapdh
34 kDa
(Mtbp band intensity
normalize to Gapdh)

Supplementary Figure S1

b

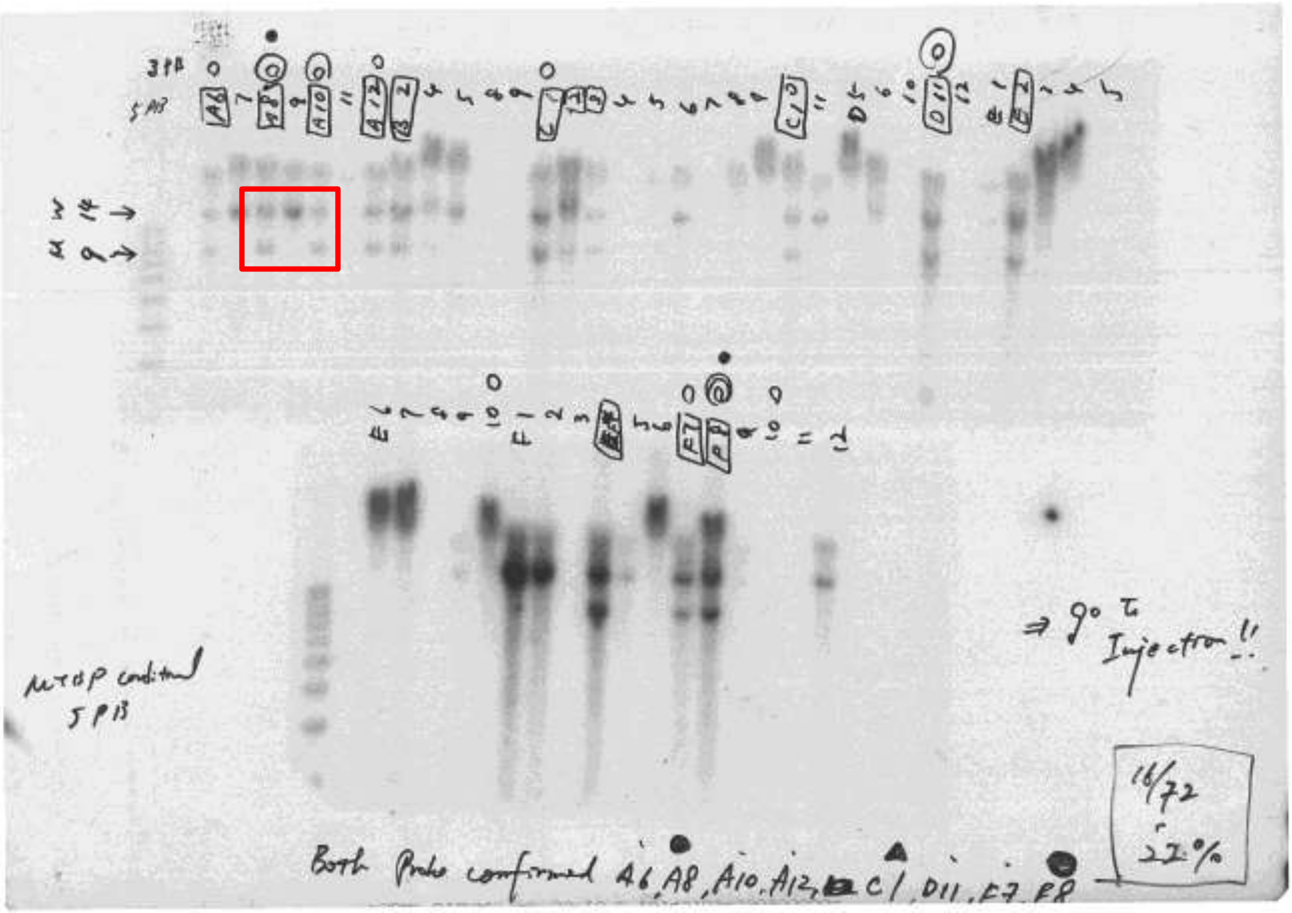

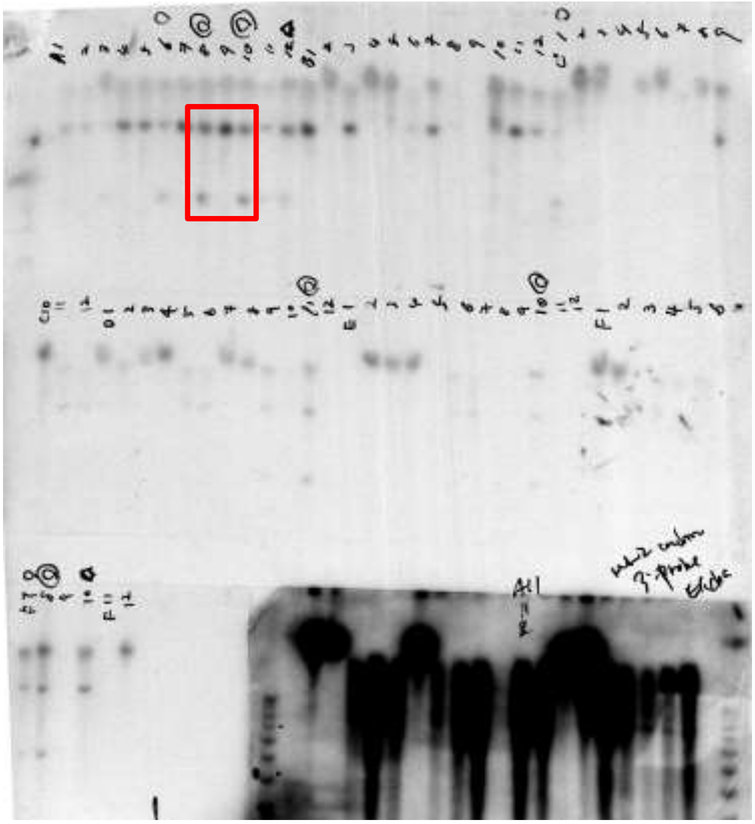

Supplementary Figure S1

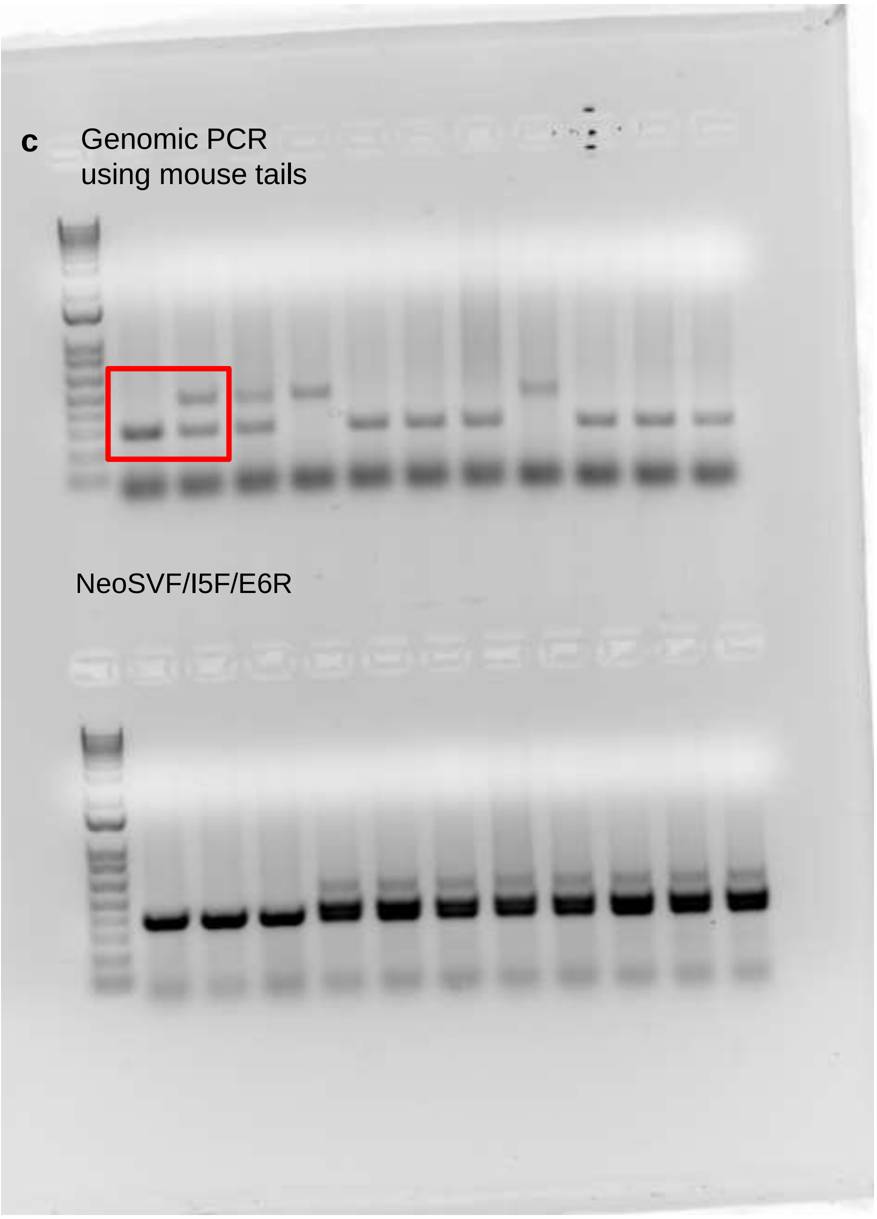

Supplement: Supplementary file 1 [file cancers-15-04596-s001.zip › Orginal images with western blots.pdf]
