# Supplementary material for: Characterization of an Mtbp Hypomorphic Allele in a Diethylnitrosamine-Induced Liver Carcinogenesis Model
Source: Cancers (Basel). 2023 Sep 16;15(18):4596. doi: 10.3390/cancers15184596 (PMC10526184; doi:10.3390/cancers15184596)
Supplement: Supplementary file 1 [file cancers-15-04596-s001.zip › Supplementary Figure S1.pdf]

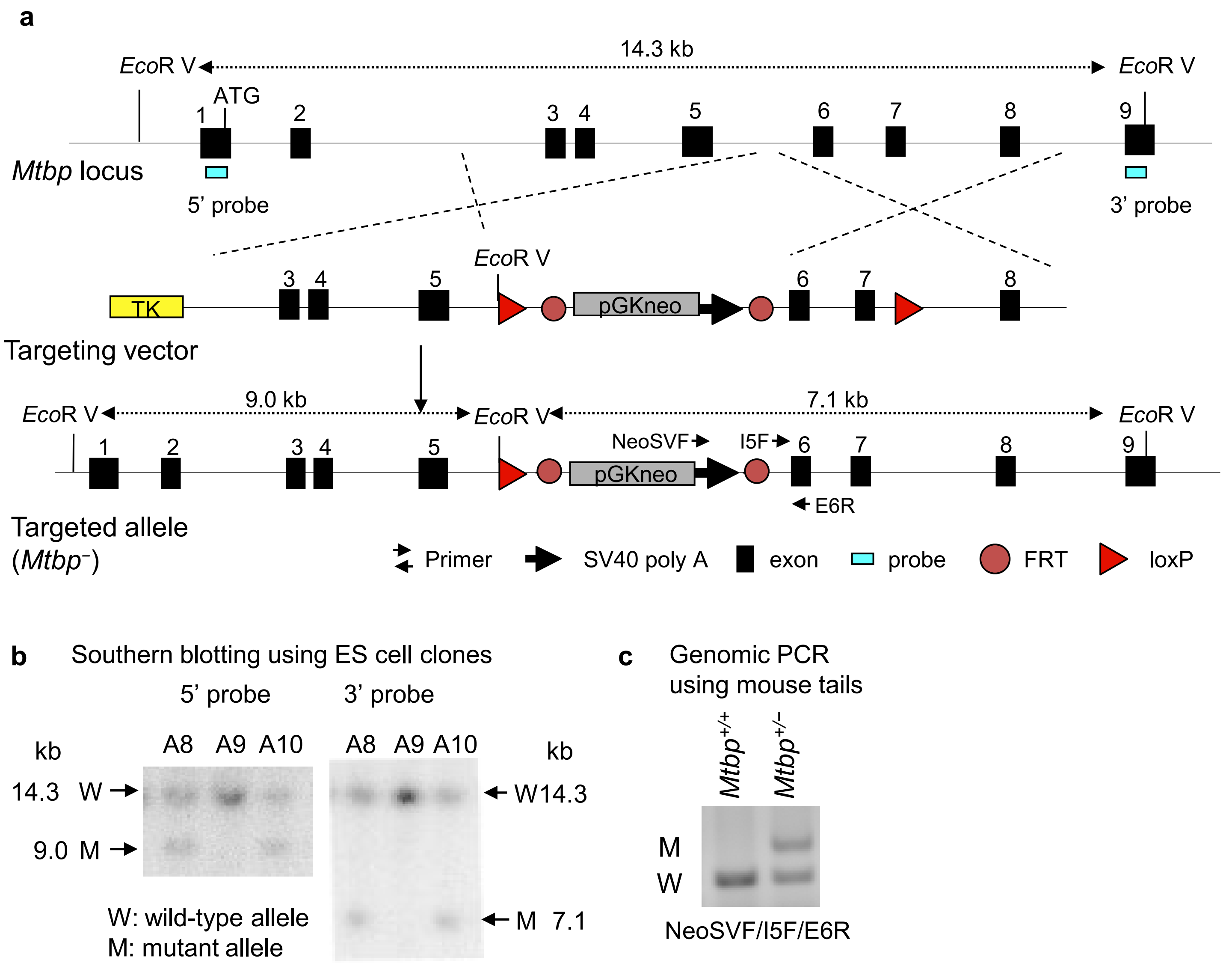

Supplementary Figure S1. Generation of mice carrying a *Mtbp* null (Mtbp−) allele. (a) Genomic organization of the murine *Mtbp* gene, a targeting vector, and a targeted allele. The *pGKneo-SV40 poly(A)* cassette was inserted into the intron 5 in the same direction as the *Mtbp* gene to block the transcription of *Mtbp.* (b) Southern blotting following *Eco* RV restriction enzyme digestion of the genomic DNA from ES cell clones (#A10, C9, A12) using the 5' and 3' probes set in exon 1 and exon 9, respectively. (c) Genomic PCR using the genomic DNA from *Mtbp*$^{+/+}$ and *Mtbp*$^{+/-}$ mice with primers of NeoSVF, I5F, and E6R, showing successful germline transmission.
